# Prognostic value of preoperative serum lactate dehydrogenase levels for resectable gastric cancer and prognostic nomograms

**DOI:** 10.18632/oncotarget.9459

**Published:** 2016-05-19

**Authors:** Zi-Xian Wang, Lu-Ping Yang, Miao-Zhen Qiu, Zhi-Qiang Wang, Yi-Xin Zhou, Feng Wang, Dong-Sheng Zhang, Feng-Hua Wang, Yu-Hong Li, Rui-Hua Xu

**Affiliations:** ^1^ Department of Medical Oncology, Sun Yat-Sen University Cancer Center, State Key Laboratory of Oncology in South China, Collaborative Innovation Center for Cancer Medicine, Guangzhou, China; ^2^ Faculty of Medical Sciences, Sun Yat-sen University, Guangzhou, China; ^3^ Department of VIP Region, Sun Yat-Sen University Cancer Center, State Key Laboratory of Oncology in South China, Collaborative Innovation Center for Cancer Medicine, Guangzhou, China

**Keywords:** gastric cancer, lactate dehydrogenase, D2 lymphadenectomy, prognosis, nomogram

## Abstract

The present study aimed to evaluate the prognostic significance of preoperative serum lactate dehydrogenase (SLDH) levels for resected gastric cancer and construct prognostic nomograms for risk prediction. The study cohort consisted of 619 patients with D2-resected gastric cancer. The relationship of SLDH levels with clinicopathological features and clinical outcomes was evaluated. Prognostic nomograms were created using identified prognosticators to predict 3-year overall survival (OS) and 3-year disease-free survival (DFS), and bootstrap validation was performed. High SLDH levels were correlated with old age but not depth of invasion or lymph node metastasis. When assessed as a continuous variable, high SLDH levels were independently associated with poor OS and DFS. Internal validation of the developed nomograms revealed good predictive accuracy (bootstrap-corrected concordance indices: 0.77 and 0.75, respectively for prediction of OS and DFS). The preoperative SLDH levels, an identified unfavorable prognosticator, were incorporated into nomograms along with other clinicopathological features to refine the prediction of clinical outcomes for patients with D2-resected gastric cancer.

## INTRODUCTION

Despite the decrease in its incidence and improvements in prognosis, gastric cancer remains the fifth most common malignancy and ranks third in terms of fatality among cancers worldwide, [1] with an especially high incidence in Eastern Asia. [2] Surgical resection is the only possible curative method for gastric cancer, especially for patients with early-stage disease, [3] but because of the high rate of postsurgical recurrence, patients with locally advanced tumors have a rather poor prognosis. [4].

Traditionally, gastric cancer outcomes are predicted on the basis of the TNM staging system, which involves tumor invasion depth, lymph node metastasis, and distant metastasis. However, other clinicopathological factors (e.g., tumor size and Lauren's classification) that may be associated with prognosis are not considered in the TNM staging system. [5, 6] Further, there is an increasing need to develop more reliable biomarkers to refine the prediction of outcomes for gastric cancer patients. [7].

Previous studies have found a high rate of glucose uptake and lactate production in tumors. [8] According to the Warburg effect, cancer cells conduct anaerobic metabolism rather than aerobic metabolism to produce most of their energy, even under normoxic conditions. On the other hand, hypoxia, a characteristic feature of solid tumors, can facilitate the process of glycolysis as well as cancer proliferation. [9] Thus, in the process of converting glucose to lactate, which is regulated by the lactate dehydrogenase (LDH), cancer cells may protect themselves better from oxidative stress, avoid mitochondria pathway apoptosis, [10] and maintain a higher proliferation rate. Serum LDH (SLDH) levels are known to be an unfavorable prognosticator in many kinds of malignancies, as has been reported in patients with melanomas, [11] lymphoma, [12] myeloma, [13] gastrointestinal malignancies, [14–21] head and neck cancers, [22] lung cancer, [23] breast cancer, [24] renal cancer, [25] prostate cancer, [26] etc. However, only two of these studies are aimed at gastric cancer, and both include patients with advanced gastric cancer. [15, 21, 27] Therefore, studies regarding the prognostic value of SLDH in patients with resectable gastric cancer remain scarce, and preoperative SLDH might be identified as an inexpensive and accessible prognosticator for these patients.

In the present study, we investigated the prognostic significance of preoperative SLDH levels in patients with resected gastric cancer after D2 lymphadenectomy and incorporated these levels into nomograms for predicting the overall survival (OS) and disease-free survival (DFS) among these patients.

## RESULTS

### Patient characteristics

Table 1 summarizes the patient characteristics. The cohort consisted of 619 patients (409 men and 210 women) with Stage IB–IIIC gastric cancer, 111 (17.9%) of whom had tumors at the gastroesophageal junction. The mean patient age was 57.9 ± 11.7 years. All patients underwent D2 lymphadenectomy; 433 (69.8%) were found to have T3/4-stage disease, and 400 (64.7%) had LN metastasis. More than 15 lymph nodes (LNs) were retrieved from 478 (77.2%) patients and more than 25 LNs from 293 patients (47.3%). Further, 414 (66.8%) patients received adjuvant chemotherapy. The median follow-up time for survivors was 29.1 months (inter-quartile range, 20.7–37.9 months). During follow-up, 161 (26.0%) patients developed locoregional/distant recurrence and 102 (16.6%) died.

**Table 1:** Patients' clinicopathologic characteristics

Clinicopathologic Characteristic	Mean or No. of patients	SD or %
**Age (years)**	57.9	11.7
≤ 60	339	54.8%
> 60	280	45.2%
**Gender**		
Male	409	66.1%
Female	210	33.9%
**Preoperative SLDH (U/L)**	159.6	33.1
≤ 245	611	98.7%
> 245	8	1.3%
**Tumor Size (cm)**	4.0	2.3
**Tumor Location**		
GEJ	111	17.9%
Non-GEJ	508	82.1%
**Differentiation**		
Moderate/high	133	21.5%
Poor/low	486	78.5%
**Lauren's Classification**		
Diffuse	303	48.9%
Intestinal	232	37.4%
Mixed	84	13.6%
**HER2 Status**		
Negative	553	89.4%
Positive	66	10.6%
**Lymphovascular Invasion**		
Yes	170	27.4%
No	449	72.6%
**Perineural Invasion**		
Yes	248	40.0%
No	371	60.0%
**pT**		
T1	103	16.7%
T2	83	13.4%
T3	296	47.7%
T4	137	22.1%
**MLN**	4.9	7.0
N0	219	35.3%
N1	125	20.2%
N2	117	18.9%
N3	158	25.6%
**THN**	25.0	11.6
≤15	141	22.8%
>15	185	29.9%
>25	293	47.3%
**LNR**	0.2	0.2
**Adjuvant Chemotherapy**		
Yes	414	66.8%
No	205	33.2%

Abbreviations: SD, standard deviation; SLDH, serum lactate dehydrogenase; GEJ, gastroesophageal junction; pT, depth of invasion; MLN, metastatic lymph node; THN, total harvested lymph nodes; LNR, lymph node ratio.

### Relationship between SLDH levels and clinicopathological factors in gastric carcinoma

The mean and median preoperative SLDH levels were 159.6 U/L and 157.2 U/L, respectively. High SLDH levels (>245 U/L) were noted in only 8 (1.3%) patients.

The correlation between SLDH level and other clinicopathologic factors is summarized in Tables 2 and 3. No significant differences were found in the SLDH levels depending on gender, tumor location, tumor size, tumor differentiation, Lauren's classification, HER2 status, pathologic T stage (pT), N stage, lymphovascular invasion, perineural invasion, or receipt of adjuvant chemotherapy. A high SLDH level was found to be correlated with older age (r = 0.228, p < 0.01), but no correlation was found between SLDH level and tumor size, metastatic lymph nodes (MLN), total harvested lymph nodes (THN), or lymph node ratio (LNR).

**Table 2:** Correlation between SLDH levels and other continuous clinicopathological factors

Correlation	r	P^*^
SLDH vs. age	0.23	< 0.01
SLDH vs. tumor size	−0.08	0.35
SLDH vs. MLN	−0.02	0.63
SLDH vs. THN	−0.01	0.87
SLDH vs. LNR	−0.02	0.55

Abbreviations: SLDH, serum lactate dehydrogenase; MLN, metastatic lymph node; THN, total harvested lymph node; LNR, lymph node ratio; r, Spearman rank correlation coefficient.

* Measured using the Spearman rank correlation test.

**Table 3:** Correlation between SLDH levels and other categorical clinicopathological factors

Variable	SLDH U/L		P
	mean ± SD	median (IQR)	
**Age (years)**			< 0.01^a^
<60	154.4 ± 32.9	150.7 (131.9–172.2)	
≥60	165.9 ± 32.5	163.25 (142.1–187.6)	
**Gender**			0.61^a^
Male	159.6 ± 34.4	156.2 (136.3–179.9)	
Female	159.6 ± 30.7	159.05 (139.4–177.8)	
**Tumor Location**			0.61^a^
GEJ	161.5 ± 29.7	158.4 (138.1–180.1)	
Non-GEJ	159.4 ± 33.8	156.9 (137.6–178.9)	
**Differentiation**			0.41^a^
Moderate/high	157.8 ± 30.7	153.2 (135.2–177.6)	
Poor/low	159.4 ± 33.5	156.8 (137.5–178.9)	
**Lauren's Classification**			0.17^b^
Diffuse	157.6 ± 35.3	155.05 (134.4–178.7)	
Intestinal	160.3 ± 30.6	157.7 (138.4–178.8)	
Mixed	164.8 ± 32.2	161.95 (144.4–183.1)	
**HER2 Status**			0.58^b^
Negative	157.8 ± 34.0	155.95 (136.0–178.8)	
Positive	163.5 ±22.1	164.3 (148.1–180.6)	
**Depth of invasion**			0.97^a^
T1&T2	158.1 ± 29.6	158.2 (139.1–177.9)	
T3&T4	160.3 ± 34.6	156.5 (136.7–180.2)	
**Nodal status**			0.60^a^
Node-negative	160.0 ± 31.2	159.25 (138.4–178.4)	
Node-positive	159.5 ± 34.3	155.9 (136.6–179.4)	
**Lymphovascular Invasion**			0.52^a^
Yes	159.8 ± 33.7	154.3 (136.5–180.6)	
No	159.6 ± 33.1	158.2 (137.9–178.6)	
**Perineural Invasion**			0.72^a^
Yes	160.5 ± 31.1	158.5 (137.7–180.6)	
No	158.7 ± 34.3	156.5 (137.0–177.7)	
**Adjuvant Chemotherapy**			0.62^b^
Yes	158.9 ± 34.4	157.2 (136.8–178.8)	
No	159.0 ± 30.2	154.8 (136.1–177.9)	

Abbreviations: IQR, interquartile range; SLDH, serum lactate dehydrogenase; SD, standard deviation; GEJ: gastroesophageal junction; pT, depth of invasion; MLN, metastatic lymph node.

a Mann-Whitney *U* test;

b Kruskal-Wallis test

### Survival analysis

The 3-year OS and 3-year DFS for the cohort were 80.2% and 70.0%, respectively. Univariate analysis showed that the SLDH level did not significantly affect OS or DFS when treated as continuous variable (OS: p = 0.087; DFS: p = 0.101) or categorical covariate divided according to the median value (log-rank: OS, p = 0.282; DFS, p = 0.189). However, in multivariate Cox regression models, the SLDH level was found to be an independent unfavorable prognostic factor for OS and DFS (OS: hazard ratio (HR) = 1.009, 95% confidence interval (CI) = 1.003–1.016, p < 0.01; DFS: HR = 1.008, 95% CI = 1.003–1.013, p < 0.01) (Tables 4 and 5). An HR of 1.009 for SLDH level as a continuous variable indicated a 50% increase in the risk of death with each 50 U/L increase in SLDH. A significant interaction was found between male gender and the SLDH level for predicting OS (p = 0.02), which indicated that the effect of SLDH level varied between the genders (Table 6); that is, high SLDH levels were associated with a greater risk of death and recurrence among male patients as compared to female patients (HR for OS: 1.012 vs. 1.000; HR for DFS: 1.009 vs. 1.006) (Tables 4 and 5), although the interaction between gender and SLDH levels was not apparent for prediction of DFS (p = 0.436).

**Table 4:** Multivariate cox regression model to predict OS

Multivariate analysis						
Variable	All		Male subgroup		Female subgroup	
	HR (95% CI)	*P*	HR (95% CI)	*P*	HR (95% CI)	*P*
**Gender**						
Female	1					
Male	12.416 (1.344–114.663)	**0.026**				
**SLDH**	1.009 (1.003–1.016)	**0.007**	1.012 (1.005–1.019)	**0.001**	1.000 (0.988–1.012)	0.971
**Tumor location**						
GEJ	1					
Non-GEJ	0.570 (0.363–0.895)	**0.015**	0.527 (0.314–0.883)	**0.015**	0.399 (0.166–0.961)	**0.041**
**pT**						
T1&T2	1	**< 0.001**	1	**0.002**	1	**0.001**
T3	3.563 (1.591–7.978)	**0.002**	3.514 (1.350–9.150)	**0.010**	2.344 (0.641–8.576)	0.198
T4	7.008 (3.045–16.131)	**< 0.001**	5.942 (2.193–16.101)	**< 0.001**	7.325 (2.001–26.816)	**0.003**
**LNR**	6.507 (3.321–12.746)	**< 0.001**	6.854 (3.109–15.107)	**< 0.001**	7.594 (2.275–25.351)	**0.001**

Abbreviations: HR, hazard ratio; SLDH, serum lactate dehydrogenase; pT, depth of invasion; LNR, lymph node ratio; GEJ, gastroesophageal junction.

Bold *P* values have statistical significance (i.e., *P* < 0.05).

**Table 5:** Multivariate cox regression model to predict DFS

Variables	Multivariate analysis					
	All		Male subgroup		Female subgroup	
	HR (95% CI)	*P*	HR (95% CI)	*P*	HR (95% CI)	*P*
**SLDH**	1.008 (1.003–1.013)	**0.002**	1.009 (1.003–1.015)	**0.004**	1.006 (0.997–1.016)	0.199
**Tumor location**						
GEJ	1		1		1	
Non-GEJ	0.528 (0.374–0.747)	**<0.001**	0.540 (0.356–0.819)	**0.004**	0.418 (0.205–0.854)	**0.017**
**pT**						
T1&T2	1	**<0.001**	1	**<0.001**	1	**0.003**
T3	2.621 (1.517–4.528)	**0.001**	2.907 (1.463–5.777)	**0.002**	2.035 (0.809–5.118)	0.131
T4	4.462 (2.511–7.926)	**<0.001**	4.430 (2.143–9.160)	**<0.001**	4.298 (1.659–11.135)	**0.003**
**LNR**	9.738 (5.711–16.603)	**<0.001**	10.054 (5.179–19.520)	**<0.001**	9.715 (3.738–25.250)	**<0.001**

Abbreviation: HR, hazard ratio; SLDH, serum lactate dehydrogenase; pT, depth of invasion; LNR, lymph node ratio; GEJ, gastroesophageal junction.

Bold *P* values have statistical significance (i.e., *P* < 0.05).

**Table 6:** Multivariate cox regression model including interaction effect between gender and sldh to predict OS

Multivariate analysis		
Variable	HR (95% CI)	*P* value
**Tumor location**		
GEJ	1	
Non-GEJ	0.518 (0.325–0.827)	0.006
**pT**		
T1&T2	1	< 0.001
T3	3.581 (1.593–8.050)	0.002
T4	7.043 (3.053–16.244)	< 0.001
**LNR**	6.621 (3.360–13.046)	< 0.001
Gender^*^	-	0.026
SLDH^*^	-	0.001
Interaction effect^*^ (Gender and SLDH)	-	0.016
**SLDH by gender**		
SLDH for female	1.000	
SLDH for male	1.012 (1.005–1.019)	0.001

Abbreviation: HR, hazard ratio; SLDH, serum lactate dehydrogenase; pT, depth of invasion; LNR, lymph node ratio; GEJ, gastroesophageal junction.

* Hazard ratios for gender and SLDH are not shown because significant interaction has been proven between these two variables, indicating that the hazard ratios for SLDH differ according to gender. Accordingly, the hazard ratios for SLDH by gender are presented.

### Predictive nomograms for OS and DFS

**Figure 1:**
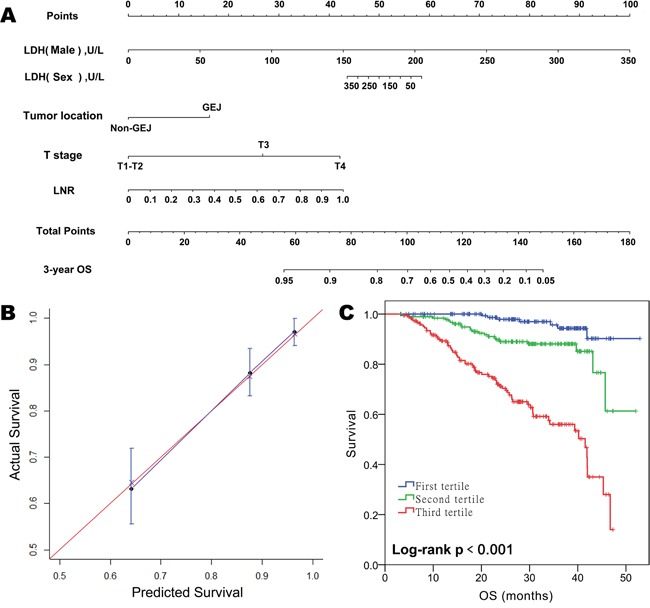
Prognostic nomogram for predicting overall survival (OS) in patients with resectable gastric cancer **A.** Predictive nomogram for OS incorporating gender, SLDH levels, and the interaction effect between these two variables along with tumor location, T stage, and LNR. For the factor “SLDH”, the points assigned should be chosen based on whether the patient was male or female. **B.** Calibration plot for nomogram-predicted OS showing close correlation with the ideal 45-degree reference line. **C.** Kaplan-Meier curves demonstrating OS in patients grouped according to the tertiles of nomogram-predicted OS. Each group represents a distinct prognosis.

**Figure 2:**
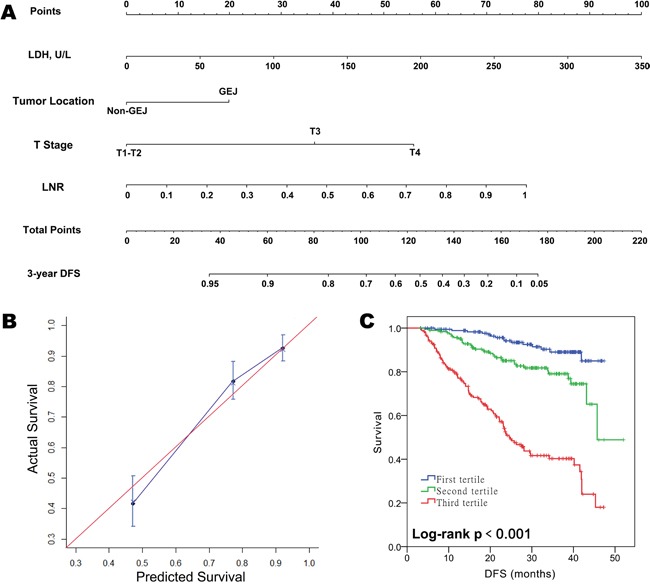
Prognostic nomogram for predicting disease-free survival (DFS) in patients with resectable gastric cancer **A.** Predictive nomogram for DFS incorporating SLDH levels along with tumor location, T stage, and LNR. **B.** Calibration plot for nomogram-predicted DFS showing close correlation with the ideal 45-degree reference line. **C.** Kaplan-Meier curves demonstrating DFS in patients grouped according to the tertiles of nomogram-predicted DFS. Each group represents a distinct prognosis.

To develop an intuitive and quantitative method to better stratify patients with different prognoses, nomograms to predict 3-year OS and DFS were developed on the basis of the final models (Figure 1A and 2A). Through RCS examination, both SLDH levels and LNR were found to have a linear effect on the HRs for OS and DFS. The association between SLDH levels and OS differed by gender. For example, a male patient with a preoperative SLDH level of 150 U/L (43 points) and pT3-stage (26 points) gastric cancer of the gastroesophageal junction (15 points) and an LNR of 0.2 (8 points) had a total of 92 points, which yielded an estimated 3-year OS of 78%. If the same patient had an SLDH level of 250 U/L, the total score would increase to 120 points, which yielded an estimated 3-year OS of 43%. In contrast, the estimation would not change drastically according to the SLDH level in a female patient with the same clinicopathologic characteristics (the total score would decrease from 100 to 97 and the estimated 3-year OS would increase from 70% to 72%).

The unadjusted concordance indices (C-indices) for OS and DFS prediction were 0.79 and 0.77, respectively, and the corresponding bootstrap-corrected C-indices were 0.77 and 0.75, indicating minimal evidence of model overfit. The nomograms showed better predictive accuracies than the 7^th^ American Joint Committee on Cancer (AJCC) TNM staging model (unadjusted C-index for OS and DFS: 0.74 and 0.73; p < 0.01 for both, Z test), and the model which incorporates tumor location, pT, LNR, but not SLDH (unadjusted C-index for OS and DFS: 0.77 and 0.75; p < 0.01 for both, Z test). The calibration plot showed excellent predictive accuracy for 3-year OS, as the prediction was similar to the ideal model (the 45-degree ideal reference line). The predictive accuracy for 3-year DFS was also good, with a less than 5% difference between the predicted and actual probabilities in each tertile (Figure 2B). By grouping the patients evenly into three subgroups according to the tertiles of nomogram-predicted OS or DFS, we found that each group had a distinct prognosis (Figure 2C). Collectively, the results showed that the nomograms were suitable for predicting OS and DFS.

## DISCUSSION

In the present study, we observed that a high preoperative SLDH level was independently associated with low OS and DFS for patients who had undergone D2 lymphadenectomy, especially male patients. Further, nomograms incorporating SLDH with other clinicopathologic factors (tumor location, pT, and LNR) showed better discrimination than the 7^th^ AJCC staging model and excellent calibration for OS and DFS prediction.

As an enzyme that participates in anaerobic metabolism, LDH may affect tumor malignancy via different mechanisms, including facilitating the proliferation, viability, and invasion capability of cancer cells, [8] and avoiding mitochondrial-mediated apoptosis. [10] In addition, several oncogenes, including HIF-1α and MYC, which are involved in upregulating genes responsible for glycolytic metabolism, angiogenesis, and cell survival, [21] were reported to be targeted by LDH. [28] Previous studies have shown that high SLDH levels are significantly associated with unfavorable prognosis in several gastrointestinal malignancies. [14–21] However, it is noteworthy that these studies were almost exclusively conducted on metastatic/non-resectable gastrointestinal tumor. To our knowledge, the prognostic role of SLDH in gastric cancer has been reported in only two studies [15, 21], and both included patients with advanced gastric cancer. The study by Zhao et al. [21] is the only previous study including patients with stage I–III gastric cancer, and it found that high preoperative SLDH levels were associated with poorer OS and DFS, consistent with our results. This previous study also found that patients with normal but relatively high SLDH levels had poorer outcomes compared with those with normal SLDH levels, which was again in line with the results of our study, in which only 8 (1.3%) patients had SLDH levels exceeding the upper limit of 245 U/L. Actually, we also divided the patients according to the median value of SLDH (157.2 U/L). As a result, normal but relatively high SLDH levels (> 157.2 U/L) were significantly associated with poorer OS (adjusted HR, 1.73; 95% CI, 1.16−2.57; p < 0.01) and DFS (adjusted HR, 1.74; 95% CI, 1.30−2.40; p < 0.01). However, Zhao et al. observed that high SLDH levels were correlated with advanced pT/pN stages but not age, while our study found that they were associated with age but not pT/pN. A reason for this could be the different distribution of clinicopathological factors between the study cohorts. For instance, the proportions of patients with stage I–II disease and those older than 60 years were higher in our cohort (51.4% and 46.8%, respectively) than those in Zhao et al.'s study (16.7% and 38.9%, respectively). In a study by Kostakis et al.,[29] the proportion of patients with stage I–II tumor was 47.5%, and SLDH levels were not found to be correlated with the T/N stage; the relationship between SLDH levels and age was not examined. Moreover, in the study by Zhao et al., [21] the proportions of patients with higher than normal LDH level were comparable among patients with Stage I−III tumor (2.4%−5.3%) but increased remarkably among patients with Stage IV tumor (14.0%). Yet, the authors did not assess the relationship between LDH and pT/pN solely among patients with Stage I−III gastric cancer. Future studies are needed to further demonstrate the relationship between LDH and locoregional cancer burden among patients with gastric cancer.

In the present study, high SLDH levels were associated with a significantly greater increase in the risk of death among male patients as compared to female patients, but the mechanisms underlying this interaction remain unclear. The male hormone androgen did not seem to be related, as older age (>60 years old) did not affect the influence of SLDH level on the risk of death or recurrence in male patients in this study (data not shown). Further, the SLDH level was found to be of prognostic value in patients with castration-resistant prostate cancer. [30, 31] Additional studies are needed to validate the prognostic role of SLDH levels in gastric cancer in male and female patients and to investigate the underlying mechanisms.

Prognostic nomograms are useful tools that allow intuitive individual risk evaluation. [32] A “point” prediction of patient prognosis is available in nomograms, and there is no need to categorize continuous variables—SLDH levels and LNR in the present study. To the best of our knowledge, ours is the first study to incorporate SLDH levels in nomograms predicting OS and DFS in gastric cancer patients who have undergone D2 lymphadenectomy. Our nomograms were more discriminative than the TNM staging. Their accuracies for prediction of OS and DFS were excellent, since nomogram-predicted survival probabilities were similar to actual survival probabilities. Kim et al. [34] constructed nomograms incorporating LNR along with patient age, gender, pT, and tumor site to predict OS and DFS and found that the nomograms had better discriminatory power than both the 7^th^ AJCC staging system and the nomograms established by the Memorial Sloan Kettering Cancer Centre. [32] The nomograms constructed in the present study had an even higher discriminatory power than those of Kim et al. [34] (C-index for OS, 0.77 vs. 0.70; C-index for DFS, 0.75 vs. 0.71), possibly because they included preoperative SLDH levels.

The present study has some limitations. First, SLDH levels may be influenced by comorbidities, such as heart, lung, or liver diseases; hypothyroidism; and anemia, which were not controlled in our study. However, the patients in our study were all eligible for surgery and survived for over 90 days after the operation, which may indicate their relatively good overall health. Second, the median follow-up was 29.1 months (inter-quartile range, 20.7–37.9 months), because of which our data were not useful for long-term survival analysis. However, since the time span was narrow and recent (2011-2013), the treatment strategies were fairly standardized as all patients underwent D2 lymphadenectomy and most received S-1/capecitabine-based adjuvant chemotherapy (67%). Third, like in the previous nomograms [32–38], adjuvant therapy was not identified as a significant prognosticator in the current nomograms. Due to many missing data regarding the cycles of adjuvant chemotherapy and the small size, and the retrospective nature, it is not practical to select candidates for adjuvant chemotherapy using the nomograms in this study. Future studies are needed to demonstrate the use of the current nomograms for patient selection. Fourth, the nomograms were developed from a Chinese cohort, and external validation using datasets from other countries is required.

In conclusion, our study identified the preoperative SLDH level as an unfavorable prognosticator in patients with D2-resected gastric cancer. SLDH levels were incorporated into nomograms along with other clinicopathologic factors in order to refine OS and DFS prediction. The nomograms were bootstrap validated, and once they are externally validated, we believe that they could be useful tools for prognosis, follow-up, and treatment.

## MATERIALS AND METHODS

### Ethics statement

All patients provided written informed consent for their information to be stored in the hospital database and used. Study approval was obtained from independent ethics committees at the Cancer Center of Sun Yat-sen University. Further, this study was undertaken in accordance with the ethical standards of the World Medical Association Declaration of Helsinki.

### Patient selection

Between December 2011 and July 2013, 847 gastric cancer patients who were diagnosed and underwent D2 lymphadenectomy at Cancer Center of Sun Yat-Sen University were identified. Patients included in the study had to meet the following criteria: (1) histologically confirmed IB–IIIC gastric adenocarcinoma; (2) histologically confirmed R0 resection; (3) follow-up data available. The exclusion criteria were as follows: (1) death within 90 days of surgery; (2) residual macroscopic or microscopic tumor, distant metastasis, or concurrent malignancies in other organs; (3) neoadjuvant chemo(radio)therapy or adjuvant radiotherapy. Finally, 619 patients remained in the study cohort.

Clinicopathological data collected for subsequent analysis included gender; age at diagnosis; preoperative SLDH level; tumor size; tumor location; degree of differentiation; Lauren's classification; HER2 status; pT; MLN and THN; LNR; presence of lymphovascular invasion; and presence of perineural invasion. HER2 status was obtained from previous pathology reports from our center. Cases with an immunohistochemistry (IHC) score of 3 or an IHC score of 2 and a positive fluorescence in situ hybridization score were considered HER2 positive.

The clinical decision to administer postoperative chemotherapy was based on the disease stage, general health, and the patient's preference. The chemotherapy regimens included single fluoropyrimidine regimens (S-1/capecitabine) and S-1/capecitabine-based combinations (S-1/capecitabine plus oxaliplatin/taxol/paclitaxel).

### Statistical analysis

Analysis was performed using SPSS 19.0 and R 3.1.2 (http://www.r-project.org/) statistical packages. Categorical variables were reported as numbers (percentage), and continuous variables as means with standard deviations. The correlation between SLDH levels and other continuous variables was assessed using the Spearman correlation analysis, and the relationship between SLDH and categorized variables was evaluated using the t-test/Analysis of Variance or Mann-Whitney U test/Kruskal-Wallis test. OS was measured between the date of surgery until death from any cause, and DFS was defined as the time from surgery to recurrence or death. Kaplan-Meier analysis and univariate and multivariate Cox regression analyses were performed to assess the association between clinicopathological factors and OS/DFS.

Previous studies have demonstrated that LNR, which accounted for both the number of metastatic and examined nodes, can compensate for the stage migration effect on survival in gastric cancer surgery [39], and improve the predictive accuracy of survival as compared with pN or number of metastatic nodes [40]. Moreover, as the number of metastatic nodes and LNR was highly correlated with each other, it would cause multicollinearity if they were included simultaneously in the Cox regression model [41]. Therefore, we used LNR instead of pN or number of metastatic nodes for model development. Restricted cubic splines (RCS) were used to examine the proportional hazards assumption and linearity assumption for continuous variables [33, 42]. An appropriate transformation was selected when a continuous variable failed to satisfy the proportional hazards assumption or linearity assumption. For model development, we began with SLDH and all other accounted variables, with or without the first-order interaction terms between SLDH and other accounted variables. The final Cox models were obtained by using backward stepwise selection of the variables (keeping only those with P < 0.05). A nomogram to predict individual survival was constructed on the basis of the final Cox model. The comparative discriminative power of the nomogram and other staging systems was assessed using the C-index: [43] the higher the C-index, the more accurate was the prognostic prediction. [34] Nomogram calibration was assessed by reviewing the plot of nomogram-predicted survival probabilities versus the Kaplan-Meier-estimated probabilities. [44] Bootstraps with 1000 resamples were used to quantify any model overfit and calculate the Kaplan-Meier-estimates.
